# Heat, Acid and Chemically Induced Unfolding Pathways, Conformational Stability and Structure-Function Relationship in Wheat α-Amylase

**DOI:** 10.1371/journal.pone.0129203

**Published:** 2015-06-08

**Authors:** Kritika Singh, Manish Shandilya, Suman Kundu, Arvind M. Kayastha

**Affiliations:** 1 School of Biotechnology, Faculty of Science, Banaras Hindu University, Varanasi, India; 2 Department of Biochemistry, University of Delhi South Campus, New Delhi, India; Aligarh Muslim University, INDIA

## Abstract

Wheat α-amylase, a multi-domain protein with immense industrial applications, belongs to α+β class of proteins with native molecular mass of 32 kDa. In the present study, the pathways leading to denaturation and the relevant unfolded states of this multi-domain, robust enzyme from wheat were discerned under the influence of temperature, pH and chemical denaturants. The structural and functional aspects along with thermodynamic parameters for α-amylase unfolding were probed and analyzed using fluorescence, circular dichroism and enzyme assay methods. The enzyme exhibited remarkable stability up to 70°C with tendency to aggregate at higher temperature. Acid induced unfolding was also incomplete with respect to the structural content of the enzyme. Strong ANS binding at pH 2.0 suggested the existence of a partially unfolded intermediate state. The enzyme was structurally and functionally stable in the pH range 4.0–9.0 with 88% recovery of hydrolytic activity. Careful examination of biophysical properties of intermediate states populated in urea and GdHCl induced denaturation suggests that α-amylase unfolding undergoes irreversible and non-coincidental cooperative transitions, as opposed to previous reports of two-state unfolding. Our investigation highlights several structural features of the enzyme in relation to its catalytic activity. Since, α-amylase has been comprehensively exploited for use in a range of starch-based industries, in addition to its physiological significance in plants and animals, knowledge regarding its stability and folding aspects will promote its biotechnological applications.

## Introduction

α-Amylase (EC 3.2.1.1; 1,4-α-D glucan glucanohydrolase), catalyzing random endohydrolysis of α-1,4 glycosidic linkages in starch and related carbohydrates with retention of α-anomeric configuration in the products [1, 2], has been a commercially significant and robust enzyme holding industrial and biotechnological importance. While heaps of facts are available on its physiological role [3], regulation of synthesis [4], catalytic properties [5] and structural features [6–9], little has been deciphered regarding the stability and folding mechanisms.

Proteins participate in nearly every chemical reaction essential for life by adopting a specific conformation, called the folded or native state, to function appropriately under *in vivo* conditions [10]. Outside of these conditions, proteins may exhibit denatured and structurally unfolded states. However, an understanding of unfolded states and the pathways through which they are achieved, allows better insight into the stability of the native state and means to prevent denaturation. Such information may lead to wider applicability in producing better enzymes for industrial purposes.

Since primary structure of a protein predisposes it towards its native conformation, it folds spontaneously during or after synthesis with precision and fidelity [11].While these macromolecules may be regarded as "folding themselves", the mechanism depends equally on the characteristics of the cytosol, including the nature of the solvent, the concentration of salts, the temperature, and molecular chaperones [11]. Thus, the protein folding problem is the physico-chemical issue of how a given protein’s conformational preferences depend on its amino acid sequence and on characteristics of its surrounding environment [10, 11]. Uncovering of this complex process might provide the ‘missing link’ in the flow of information from a gene sequence to the 3D structure of a protein and a unique insight into the way in which evolutionary selection has influenced the properties of a molecular system for functional advantage [12]. The resolution of this puzzling process of 3D architecture can prepare stronger grounds for protein structure prediction and design. However, to achieve this goal, one must investigate a large number of proteins, preferably multi-domain and multi-subunit, probing the influence of environment on their unfolding characteristics. α-Amylase suits this purpose well and is the subject of present investigation.

The protein folding pathway occurs along a funnel, narrowing down to intended folded polypeptide with minimum conformational entropy. The folding process of an unfolded protein involves a stochastic search of the many conformations accessible to a polypeptide chain based on the coexistence of many competing interactions among amino acids, which at times may not be foolproof, leading to detrimental pathological conditions [13, 14]. Protein landscape on its way, downhill, consist of regions of local minima which anchor partially folded intermediates formed during thermal or denaturant induced equilibrium unfolding and they fold rapidly by making local independent decisions first, followed by final global decision [5]. An understanding of the structural and thermodynamic properties of such intermediate states may provide insight into the factors involved in guiding the pathway of folding, as the road to the native state from the vast majority of individual non-native conformations is downhill and is different for each non-native starting conformation. Hence, understanding of the microscopic folding routes of a given chain conformation, to reach the downhill gulley that can take it to the native state and it avoids traps and hills on way (dead conformations) [15], will not only help to unravel the protein folding enigma but also provide a coherent insight into the molecular basis of the stability of a protein.

α-Amylase is an industrially significant and potent candidate in the enzyme market. Given that, it has to operate in industries under extreme environmental conditions, continuous research for this enzyme with improved characteristics (structural and functional stability) has been in place [16]. The enzyme has been the mainstay of starch and its derivative industries influencing fields of textile, paper making, bakery, food, ethanol production, fermentation, detergents and brewing/distilling industry [17]. Apart from these, α-amylase has been targeted for design of therapeutic agents against type II diabetes, obesity, hyperlipidemia and caries [2]. Amylases are also involved in anti-inflammatory reactions triggered by the release of histamine and similar substances. An amylase deficiency can include skin problems such as psoriasis, eczema, hives, insect bites, allergic bee and bug stings, atopic dermatitis, and all types of herpes. Hyperamylasemia and hyperamylasuria of varying degrees are frequently observed in patients with lung and ovarian cancers [18–20]. Therefore, understanding how structure-function relationship of a protein changes with changing environmental conditions is fundamentally important for both theoretical and applicative aspects. Present study using α-amylase provide insights into the structural and physical stabilities of this industrially vital enzyme with evidence for intermediate states in its multiple unfolding pathways that exist in a divergence from classical two-state mechanism.

## Materials and Methods

Wheat α-amylase was purified as described recently [21]. Homogeneity of purified preparation was checked by SDS-PAGE and size-exclusion chromatography (SEC). GdHCl (guanidium hydrochoride), urea, ANS (8-anilino-1-naphthalene sulfonate) and Bradford’s reagent were obtained from Sigma Chemical Co. (St. Louis, Mo, USA). All the chemicals for buffers were of analytical grade from Merck (Eurolab GmbH Damstadt, Germany). Milli Q (Millipore, Bedford, MA, USA) water with a resistance higher than 18 M*Ω* cm was used all throughout the experiments. Samples for spectroscopic measurement were centrifuged and filtered through 0.45 μm filters. Exact concentration of the protein and pH were determined before any spectroscopic measurement.

### Enzyme activity and protein estimation

Enzyme activity can be regarded as the most sensitive probe to study the changes in enzyme conformation during various treatments, because it reflects subtle readjustments at the active site, enabling very small conformational variations of an enzyme structure to be detected [22]. Enzyme activity measurements also indicate the integrity of the tertiary structure of the enzyme against denaturants. The catalytic activity of α-amylase under various conditions of pH, temperature and denaturants was estimated using starch as substrate, as described recently [21]. For acid induced denaturation studies, the enzyme was incubated in a range of pH as mentioned below for 20 h and assayed at pH 5.0. For pH optimum investigation, the assays were however performed at the respective pH at which the enzyme and substrate were incubated. To assess the influence of chemical denaturants on the enzyme activity, the enzyme was incubated at various denaturant concentrations for 20 h and assayed at pH 5.0. The enzyme was also subjected to increasing temperature and incubated at each temperature for 5 min before activity was measured at pH 5.0. For determination of temperature optima, the enzyme and substrate were incubated at a particular temperature and assayed at the same temperature.

Protein content was estimated using Bradford’s method [23], with crystalline bovine serum albumin as the standard protein.

### Circular dichroism (CD) spectroscopy

Circular dichroism (CD) measurements were performed on a pre-calibrated (with 0.1% d-10-camphorsulfonic acid solution) JASCO J-815 CD spectropolarimeter (JASCO Corporation, Hachioji-shi, Tokyo, Japan) equipped with Peltier multiple holder. Temperature was controlled and monitored with a circulating water bath (Multitemp, Pharmacia, Sweden). Conformational changes in the secondary structure of protein were monitored in the region between 190 to 250 nm with a protein concentration of 50 μg ml^-1^ (1.56 μmol) in a quartz cuvette (Starna, Hainault, Essex, England) with a path length of 1 mm. The scanning speed, band width and data pitch were set to 50 nm min^-1^, 1 nm and 0.5 nm, respectively. Three accumulation of scans were taken (within 700 HT voltage range) and averaged to get the complete spectra. The content of secondary structures (α-helix, β-sheets) of the protein was obtained from K2D2 server by providing the far-UV ellipticity data points in the range of 190–240 nm.

### Fluorescence spectroscopy

Fluorescence spectra were collected on Varian Cary eclipse spectrofluorimeter (Varian, Inc. Palo Alto, CA, USA) equipped with Varian Cary temperature controller (Peltier multiple holder). Tryptophan was selectively excited at 292 nm, whereas for both the tryptophan and tyrosine fluorescence of α-amylase, the excitation wavelength was 278 nm. The emission spectra were collected between 310 to 450 nm using microfluorimeter cell quartz cuvette (Varian, Palo Alto, CA, USA) with a path length of 10 mm. Scan speed was set to 100 nm min^-1^ using response time of 1 sec. Slit width for both emission and excitation were 10 nm. The protein concentration used for all fluorescence experiments was 10 μg ml^-1^. All fluorescence spectra were corrected for background intensity as measured with pure buffer.

The extent of exposure of hydrophobic surface in the protein was measured by its ability to bind with the ANS fluorescent dye [24]. A stock solution of ANS was prepared in methanol and the dye concentration was measured using an extinction coefficient (ε) of 5000 M^-1^ cm^-1^ at 350 nm [25]. For estimation of hydrophobic surface of protein, the sample was incubated with a 100-fold molar excess of ANS at room temperature in the dark. The fluorescence was measured after 30 min with excitation at 380 nm and emission between 400 to 600 nm.

### Acid and temperature induced denaturation

Acid denaturation of α-amylase was carried out as a function of pH using glycine-HCl (pH 2.0–3.0), sodium acetate (pH 4.0–6.0), Tris acetate (pH 7.0–8.0) and sodium tetraborate (pH 9.0–10.0). After addition of protein, the final concentrations of all buffers were adjusted to 20 mM. A stock solution of the protein was added and mixed to the appropriate buffer and was incubated for 20 h at room temperature (25±1°C).

Heat induced denaturation of α-amylase was performed as a function of increasing temperature using Peltier temperature controller in 20 mM sodium acetate buffer, pH 5.5. Protein samples were incubated at the desired temperature for 5 min before ellipticity (CD) and fluorescence emission measurements were performed.

### Chemical induced denaturation

Chemical denaturation of the enzyme was performed with increasing concentrations of the denaturants at pH 5.5. The protein sample was incubated at a desired denaturant concentration for approximately 20 h at room temperature (25±1°C) to attain equilibrium.

In all unfolding experiments, the extent of denaturation was expressed in terms of fraction unfolded (*α*_*i*_) at i^th^ concentration of denaturant and can be calculated from the Eq (1) [26, 27]

$$\alpha_{i}=\left({\left[\theta \right]}_{i}-{\left[\theta \right]}_{f}\right)⁄\left({\left[\theta \right]}_{u}-{\left[\theta \right]}_{f}\right)$$ (1)

where [*θ*]_*f*_ is the ellipticity of the molecule when it is fully folded and [*θ*]_*u*_ is the ellipticity of the molecule when it is fully unfolded.

The unfolding constant for a monomeric protein was calculated according to the Eq (2) given by Greenfield [26] and Tripathi [27]

$${\text{K}}_{ui}=\alpha_{i}⁄\left(1-\alpha_{i}\right)$$ (2)

The free energy of unfolding of a protein at any given concentration of denaturant (ΔG_*ui*_) can be evaluated from Eq (3):

$$\Delta {\text{G}}_{ui}=-2.303\;\text{RT}\;\text{log}\;{\text{K}}_{ui}$$ (3)

where R is the gas constant (1.98 cal mol^-1^K^-1^) and T is the absolute temperature [27]. Free energy of protein unfolding (ΔG_0_) was determined from a plot of ΔG_*ui*_ (y) as a function of denaturant (x) where y intercept equals to the ΔG_0_. A half-Chevron plot was obtained from the values of unfolding kinetics rate constant (K_obs_) plotted against denaturant concentration.

### Homology modeling and sequence alignment

Wheat α-amylase three-dimensional structure was homology modeled *in silico* using Swiss Model server (http://swissmodel.expasy.org/) with rice α-amylase as the template with 92% query coverage and 68% sequence identity. The aromatic amino acid residues were visualized using Discovery Studio 3.0 and highlighted in the model. The amino acid sequence of wheat α-amylase was aligned with those from *Hordeum vulgare*, *Bacilus licheniformis* and *Aspergillus oryzae* using Clustal W (www.ebi.ac.uk/Tools/msa/clustalw2/).

## Results and Discussion

Limited mechanistic understanding of the innate ability of proteins to fold into their native, functional form from their one dimensional unit, have plagued researchers for past several decades, although several theories have emerged about this remarkable biochemical event based on studies of small, monomeric, single-domain proteins [28–30]. One such theory is the widely accepted two-state protein folding process for many proteins [31, 32], which needs constant re-evaluation, especially for larger multi-domain proteins. Based on the co-existence and interplay of various physiochemical forces, the folding of multi-domain proteins, however, was proposed to proceed in discrete steps corresponding to the folding of individual domains that assemble to form native structure with minimum free energy and deviation from two-state model [33]. The conformational transition of such proteins is given by a specific trajectory or bundle of trajectories on energy landscape being dictated by the primary sequence as well as the environment surrounding it [34].

**Fig 1:**
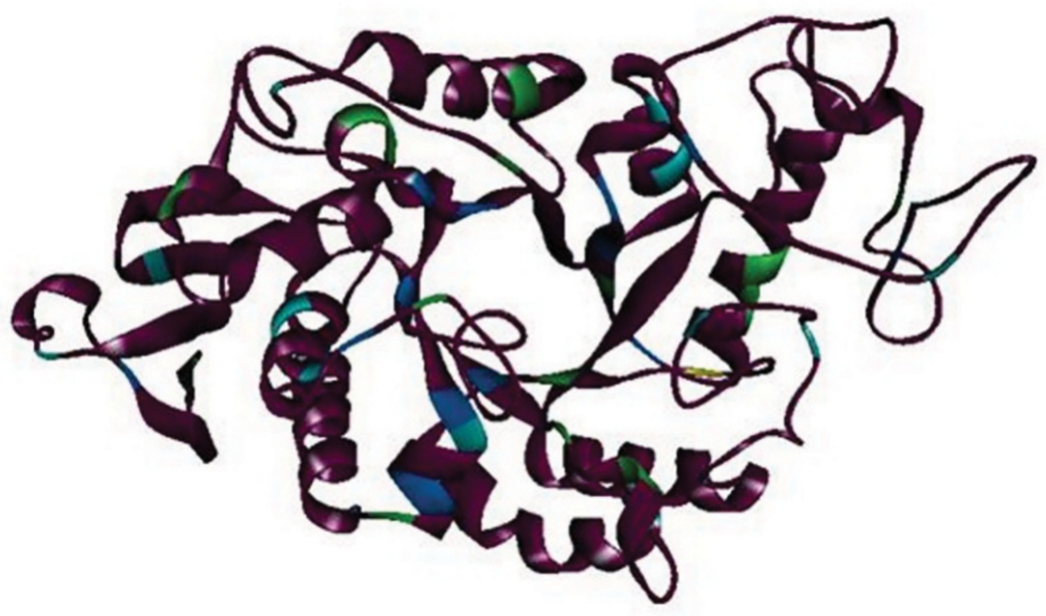
Homology model of wheat α-amylase, with aromatic residues highlighted in the following colors: tryptophan—green; tyrosine—cyan; phenylalanine—light blue. The enzyme displayed 13 tryptophan, 15 tyrosine and 16 phenylalanine side chains.

As the conformational stability of the native protein is a function of external variables, such as temperature, pH, ionic strength, and solvent composition, a quantitative analysis of their role in the structure formation is a requisite for the description of the forces that are responsible for the conformational stability, which in turn can provide insight into the molecular structure of the enzyme and ways to improve it [34]. A simple method for such studies involves the monitoring of conformational changes that are caused upon perturbation of a protein molecule by various agents, such as acid, temperature, GdHCl or urea. Since elevated temperature and high concentrations of chemical denaturants disrupt the secondary components as well as higher ordered functional structure, therefore, the present study aims to understand the structure-function relationship and folding-unfolding mechanism of α-amylase under specific environmental and denaturant condition, using circular dichroism, fluorescence spectroscopy and enzyme activity measurement. For a correlation of structure and function, the three-dimensional structure of wheat α-amylase was built *in silico* (Fig 1). It is evident that the enzyme is a member of the α+β class of proteins, as expected. The protein also displayed a large number of aromatic amino acid side chains (color coded) distributed throughout its structural parts, which can conveniently report local perturbations in conformation and three-dimensional structure *via* intrinsic fluorescence measurements (S1 Text). The enzyme activity measurements can also report changes in native tertiary structure while far-UV CD probes the perturbations in secondary structure of the enzyme.

**Fig 2:**
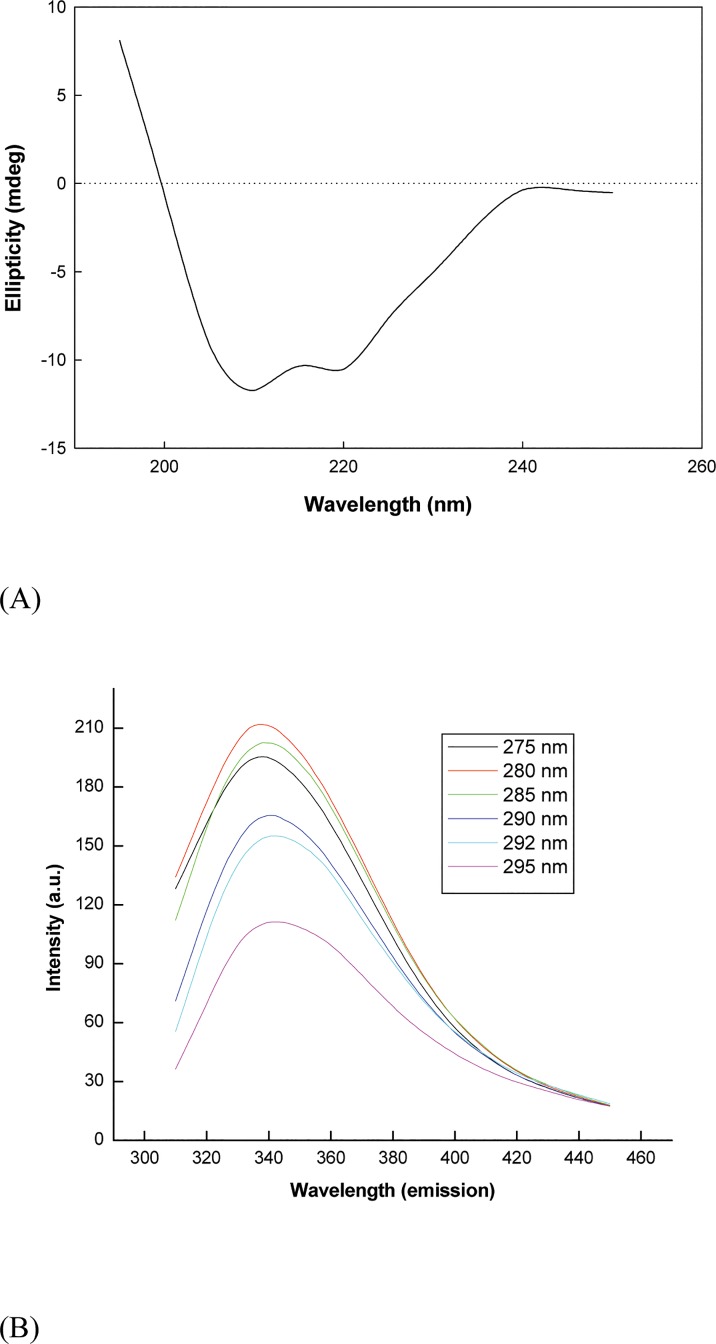
Spectroscopic properties of α-amylase. (A) Native far-UV CD spectrum and (B) Fluorescence emission spectra at different excitation wavelengths of α-amylase reveal similar emission maximum (tryptophan, 340 nm) with higher energy emission at lower excitation wavelengths (as both tyrosine and tryptophan are excited) whereas lower emission at higher wavelength (only tryptophan being excited).

α-Amylase is a monomeric protein from wheat with a native molecular mass of 32kDa. The enzyme was found to be quite stable in acetate buffer (20 mM, pH 5.5) over weeks as monitored by enzyme activity and SDS-PAGE [21]. Hence, thermal and chemical denaturation studies were carried out in 20 mM acetate buffer (pH 5.5).The enzyme was shown to belong to α+β class of protein at pH 5.5, as exhibited by its characteristic native far-UV spectrum measured by circular dichroism (Fig 2A) [35], as also evident in its *in silico* model (Fig 1). Secondary structural content was determined from the CD spectrum of native protein using K2D2 server, which revealed 15% α-helices and 26% β-sheets at 20 mM acetate buffer (pH 5.5). It was found to be similar to α-amylase secondary structural content from *Cryptococcus flavus* (α-helices = 9.5%; β-sheets = 32.4%) [36]. These results indicate the presence of substantial segments (59%) of the enzyme which are devoid of regular secondary structure, as also evident in the *in silico* model which displays substantial loops (Fig 1). The lower content of regular secondary structure could be attributed to the high glycine content of α-amylases [37]. This in turn indicates the high flexibility and conformational plasticity of the plant enzyme. It has been reported before that mammalian α-amylases exhibit a highly flexible glycine-rich loop, while it is absent in insect α-amylases [38]. Our investigation of structural property of wheat α-amylase here indicates that the plant amylases would be similar to mammalian amylases and unlike the insect amylases. Since the glycine loop allows for “trap-release” mechanism for substrates, this allows design of specific inhibitors against insect amylases feasible to protect crop with the expectation that these inhibitors would not inhibit either mammalian or plant amylases [39].

To determine the intrinsic fluorescence properties, emission spectra of the native protein were obtained at varying excitation wavelengths since the protein contained multiple tyrosine and tryptophan side chains (Fig 2B). The spectra showed similar emission maximum (λ_max_ = ~340 nm), typical of tryptophan emission [40], with varying intensity at different excitation wavelengths. It thus appears that the fluorescence energy of tyrosine was effectively quenched and transferred to tryptophan side chains in the vicinity with insignificant emission maximum typical of tyrosine. At lower excitation wavelengths (278–285 nm), the intensities of emission were higher since both tyrosine and tryptophan were excited. At higher excitation wavelengths (290–295 nm), only tryptophan was selectively excited resulting in lower emission intensities. Such fluorescence properties indicate specific interactions between tyrosine and tryptophan residues, many of which must be near to each other for energy transfer, creating hydrophobic clusters. In addition, the fact that tryptophan residues can be selectively excited, allows for further investigation of protein folding mechanism in α-amylase using site-directed mutagenesis approach and fluorescence as a probe.

Fluorescence spectra provide a localized signal which helps to ascertain the extent of modifications in the local environment of the fluorescent probe upon folding/unfolding of protein conformation [41].This spectrum is determined chiefly by polarity of the environment of the tryptophan and tyrosine residues and by their specific interactions [41]. In summary, the fluorescence emission maximum suffer a red-shift when chromophores become more exposed to solvent and the quantum yield of fluorescence decreases when the chromophores interact with quenching agents, either in a solvent or in protein itself. The advantage with multiple tryptophan residues, especially if they are distributed over the entire structure, is that they act as reporters for the structural state and integrity of all regions of the molecule simultaneously maintained.

### Thermal denaturation

**Fig 3:**
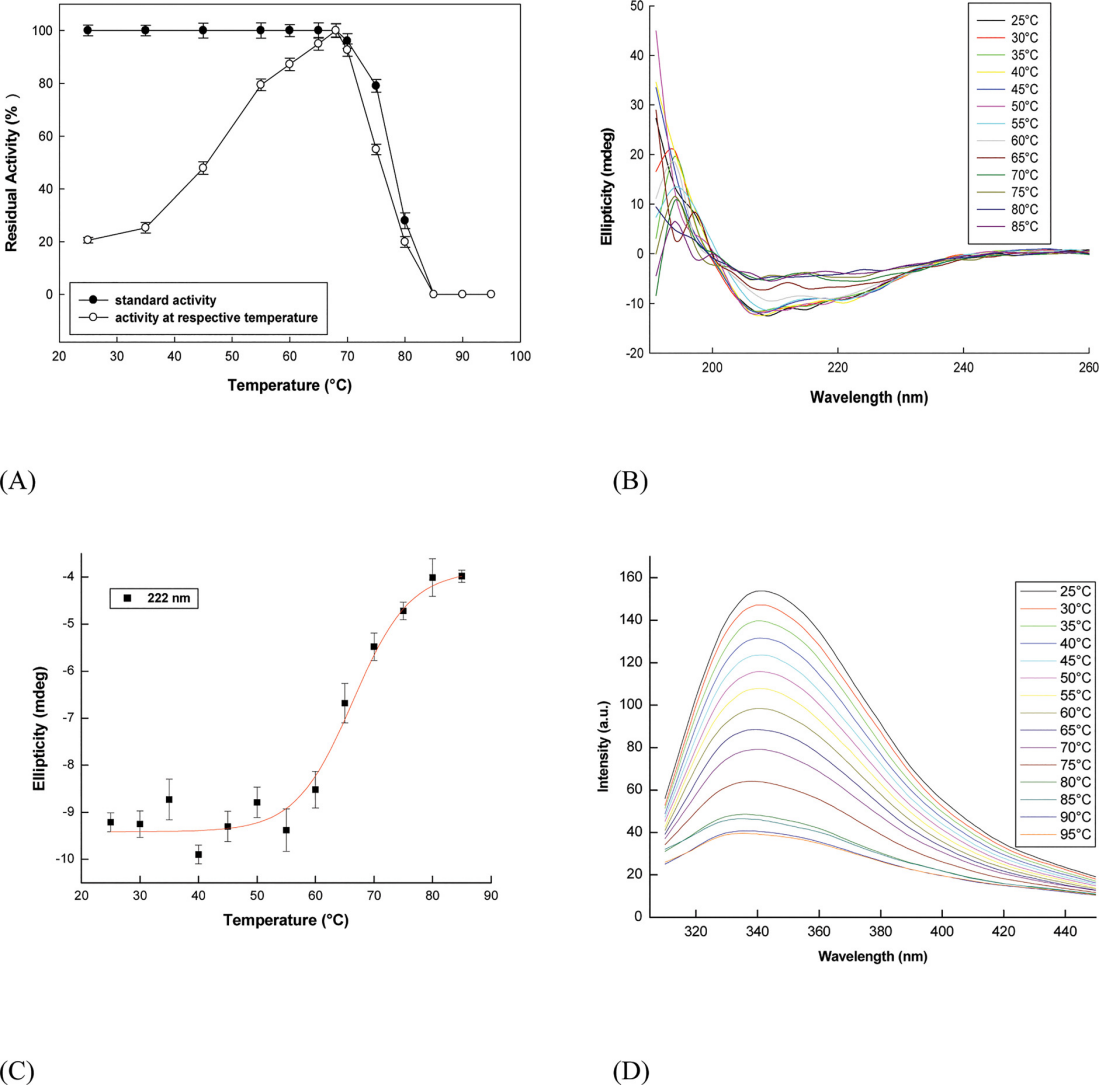
Thermal denaturation profiles of α-amylase (A) Residual enzymatic activity as a function of heat denaturation was estimated both at respective temperature of incubation (open circle) and at 68°C followed by incubation for 5 min at various temperatures (closed circle) (B) Far-UV CD spectra at various temperatures (C) Changes in ellipticity at 222 nm with temperature (D) Temperature-dependent fluorescence spectra of α-amylase.

A unique native structure for proteins is a pre-requisite for its proper functioning. The ability to build up and to keep this native and functional structure in a particular range of temperatures is an intrinsic property of the protein itself, which is determined by the amino acid sequence [42]. α-Amylase, a multi-domain enzyme well-known for its thermostability, has been the model enzyme to study and elucidate the origins and mechanisms of thermostability and thermal adaptation [43].The hydrolytic activity of α-amylase went unperturbed up to 65°C (Fig 3A) and it lost only 4% of catalytic activity at 70°C, whereas complete loss of enzyme activity was observed around 85°C.

However, α-amylase did show decrease in ellipticity (θ_222_) beyond 60°C (Fig 3B and 3C), indicating that the part of the protein that gets perturbed structurally, first (maybe the polypeptide terminals) does not immediately affect enzyme activity and must be away from the active site (catalytic domain). No significant changes in fluorescence emission maximum (λ_max_) with respect to increasing temperature was observed (Fig 3D) indicating that the fluorophores were sufficiently in polar environment in the native protein (λ_max_~340 nm also indicates the same) probably due to the presence of–OH groups of nearby tyrosine. However, a steady decrease in fluorescence intensity with increase in temperature (even below 70°C) indicates the quenching of fluorescence due to exposure to solvent or appearance of quenchers within the protein (*viz* anions). In summary, the results suggest that the enzyme is functionally and globally (structure-wise) very stable with local conformational changes upto 65–70°C. Thermally denatured wheat α-amylase did not restore maximal fluorescence intensity or enzymatic activity even after prolonged cooling, indicating irreversible denaturation. The temperature optima of 68°C(Fig 3A) also supports the fact that the tertiary structure is stable till 70°C and subsequently shows perturbation beyond this temperature.

With further increase in temperature beyond 70°C, protein probably undergoes denaturation with concomitant aggregation that leads to intrinsic irreversibility and precludes thermodynamic analysis of unfolding. The exposed hydrophobic clusters in the protein might be responsible for aggregation. For most multi-domain proteins, the thermal unfolding transitions are accompanied by an irreversible step, often related to aggregation at elevated temperatures as chemical modifications such as deamidation, cysteine oxidation, or peptide bond hydrolysis, take place once the protein is unfolded [41–44]. Hence, the analysis of thermo-stabilities in terms of equilibrium thermodynamics is not applicable if the irreversible process is fast with respect to the structural unfolding transition.

Enzyme maintained its secondary structure up to 60°C and beyond this temperature it started losing its secondary structure. This multi-domain protein at temperatures below its thermal inactivation, due to thermal fluctuations, might have led to transient unfolding of respective domains exposing the hydrophobic interior to the solvent while keeping other structural elements intact, which can prime the molecule for aggregation [45]. As a consequence of protein aggregation at higher temperatures, tryptophan residues might also remain buried within protein which limits their further exposure to solvent. Hence, not much change could be observed in emission maximum even at higher temperatures unlike the characteristic red-shift of tryptophan in polar solvent [41, 43]. The reduction in fluorescence intensity at higher temperatures could be attributed to the decrease in distance between tryptophan and specific quenching residues such as protonated carboxyl, protonated imidazole, deprotonated ε-amino groups and tyrosinate, which quench tryptophan residues effectively at elevated temperature [46].

### pH induced denaturation

**Fig 4:**
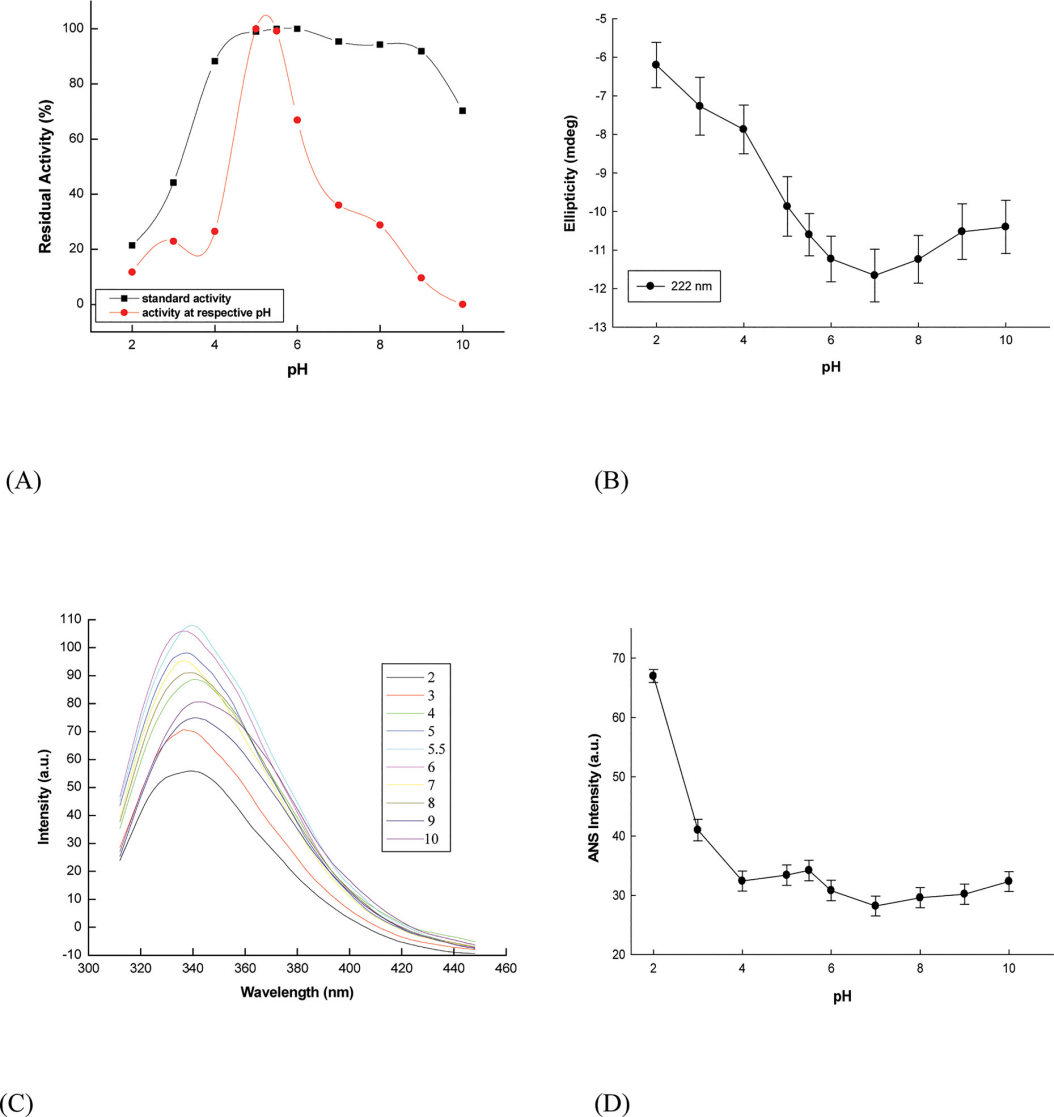
pH induced denaturation profiles of α-amylase (A) α-Amylase activity as a function of pH. The enzymatic activity was estimated both at respective pH of incubation (red) and at pH 5.0 followed by incubation at various pH (black) (**B**) Changes in ellipticity at 222 nm with pH (**C**) pH dependent fluorescence spectra of α-amylase (**D**) Fluorescence intensity of ANS binding to α-amylase at different pH.

Structural perturbations at extreme pH conditions occur, mainly, due to disruption of electrostatic interactions and hydrogen bonding, which play a significant role in protein stability [46]. The structural and functional changes in α-amylase upon changes in pH were followed by far-UV CD, fluorescence and enzyme activity measurements over a wide pH range (Fig 4). The enzyme retained structural and functional stability in the pH range of 4.0–10.0. Cooperative transition was observed during its unfolding from native state. Acidic environment did induce a significant reduction of the regular conformation, suggesting that wheat α-amylase failed to preserve its three dimensional structure at very low pH; however, unfolding was incomplete.

Enzymatic activity was estimated at various pH according to the standard assay procedure and the pH optimum was observed to be 5.0 (Fig 4A), with rapid decrease in activity in either direction [21]. Enzyme incubated in the pH range 4.0–9.0 and assayed at optimum pH (stability experiments) exhibited more than 88% recovery of hydrolytic activity, which decreases rapidly below pH 4.0 but resists denaturation to some extent above pH 9.0 (Fig 4A). At pH 2.0, the enzyme showed irreversible loss of catalytic activity (84%). However, at pH 2 the protein retained about ~50% of its secondary structural component (ellipticity at 222 nm = –6.6 mdeg while native protein ellipticity was –12 mdeg) as evident from its far-UV CD spectrum (Fig 4B). Upon excitation at 292 nm, the protein exhibited a small red-shift in fluorescence emission maximum at pH 2 and I_max_ dropped from 106 (at pH 6) to 55 (at pH 2) (Fig 4C), indicating local conformational changes but incomplete unfolding. As observed above, comparisons of the plots in Fig 4 indicate that while the structure of α-amylase is more compact near to neutral pH, the enzymatic activity is maximal at pH 5, indicating that slightly perturbed conformational changes in some domains of the enzyme allow for higher enzyme activity. The spectroscopic properties also indicate that pH 2 induces intermediate state in α-amylase, which was probed by ANS fluorescence.

ANS is essentially non-fluorescent in aqueous solution, whereas its emission intensity increases significantly in a hydrophobic environment [47]. Hence, exposure of any hydrophobic regions, buried inside the enzyme in the native state, on acid unfolding, was monitored using ANS dye. ANS binding did not show any significant change in fluorescence intensity in the range of pH 4.0–10.0, but increased about 2.4 fold at pH 2.0 and 1.5 fold at pH 3.0 (Fig 4D). Thus on decreasing the pH below 4.0, the protein probably unfolded and became less compact with the exposure of hydrophobic residues as evident from ANS binding. These observations suggest the presence of a large number of solvent-accessible non-polar clusters in the protein molecule at pH 2.0 as the ANS binds to hydrophobic surfaces on the protein with greater affinity.

The protein conformation at pH 2.0 retained significant secondary structure with irreversible loss of activity and opening of tertiary packing, as evident from intrinsic and extrinsic fluorescence as well as activity measurements, putatively suggesting it to be an intermediate state during unfolding. This intermediate state is often called the molten globule and contains reduced secondary structural elements and loose tertiary level interactions [48]. Also, the existence of hydrophobic surface pockets is a good index for molten globule state. Several proteins in this state have been shown to be sticky and prone to aggregation [49].This is the first report of pH induced unfolding of any plant α-amylase, and further studies are required to get additional insights of pH induced unfolding kinetics.

### Chemical denaturation

**Fig 5:**
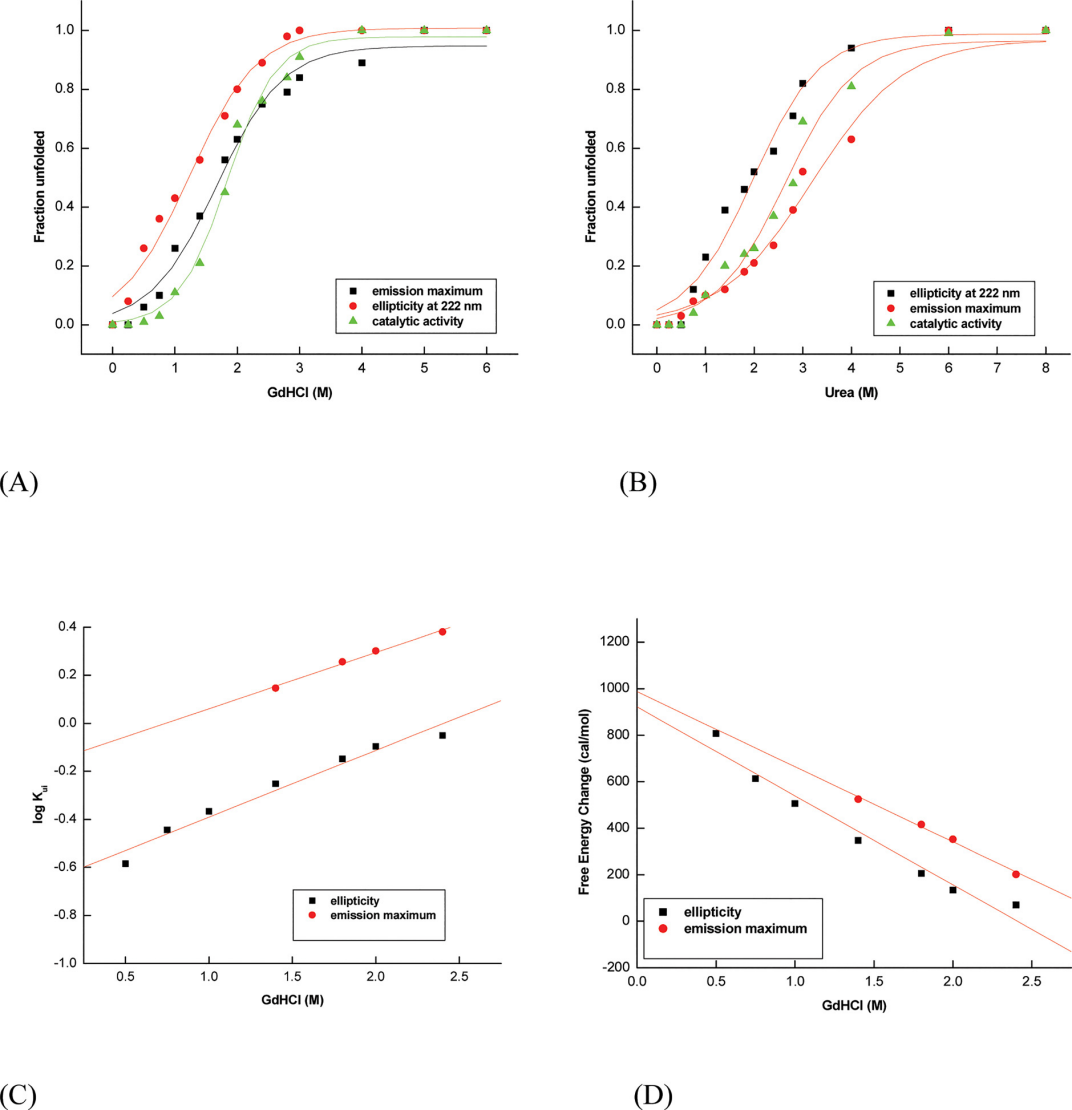
Chemical induced conformational changes of α-amylase (A) and (B) represents GdHCl and urea mediated transitions of protein unfolding, respectively (C) Unfolding kinetics rate constants (Half-Chevron plot) against GdHCl (D) Free energy of unfolding. Intercepts at y axis returns log K_obs_ and ΔG_0_, respectively.

The protein was chemically denatured with different concentration of GdHCl or urea at pH 5.5 for 20 h, prior to spectral or enzyme activity measurements. These chaotropes are known to affect the protein structure by disturbing the electrostatic interactions present on its surface [50–52] whereby they not only destabilize the native state, but also protect the protein in their unfolded state from aggregation [53]. GdHCl induced denaturation of α-amylase showed cooperative transitions that are irreversible, non-coincidental and sigmoidal (Fig 5A). The disruption of secondary structure begins below 0.5 M GdHCl, as evident from decrease in ellipticity, whereas fluorescence and enzymatic activity does not significantly alter up to this point. This loosening of the tight folding of native structure, leading to decrease in protein’s secondary structure, as also observed above, occurs as a separate process preceding the exposure of the aromatic side chains to the solvent and active site perturbation [54]. The intact catalytic activity indicates that the secondary structural perturbation occurs in a domain outside from the catalytic domain, which might not be necessary for maximal activity of the enzyme.

Previously, there have been reports on GdHCl driven unfolding of α-amylase being governed by two-state transition (*F/N*↔*U*) without any indication of intermediates in the unfolding process [55–57]. Contrary to two-state folding mechanism, in the present study, non-coincidental transitions for α-amylase do indicate the presence of intermediates, which might be transient resulting from perturbations of domains at specific intervals rather than simultaneous transitions. Soybean α-amylase which followed simple two-state unfolding under GdHCl induced denaturation at pH 7.4, underwent complete reversible unfolding with transition midpoint at 0.73 M GdHCl. However, denatured soybean α-amylase refolded back to native state in the presence of 7M glycerol in refolding buffer so as to avoid the aggregation of the side chain, which was practically minimized under these conditions [57]. The refolded enzyme was found to be indistinguishable from native enzyme, in terms of biochemical and biophysical characteristics. It is evident from our studies that such phenomena need to be investigated at a pH where enzyme activity is maximal (which is 5–5.5 for wheat α-amylase) for a complete insight into the protein folding process.

Urea also showed complete irreversible unfolding of α-amylase at 8 M concentration where again ellipticity decreases and secondary structure falls off prior to changes in enzyme activity and emission maximum (Fig 5B). The enzyme showed resistance to unfolding till 1.4 M urea concentration as there was not much change in its activity and emission maximum (Fig 5B). Urea mediated unfolding was found to be cooperative and transitions were non-coincidental and sigmoidal, with non-coincidental transition indicating the probable existence of an intermediate state in unfolding.

The equilibrium unfolding of α-amylase by various denaturants revealed its stability, with the enzyme retaining its structural integrity and hydrolytic activity in the presence of 1.2 M GdHCl as well as in the presence of 2.0 M urea, indicating the high rigidity of the molecule. A significant red-shift of 15 nm (340 nm to 355 nm) in the wavelength emission maximum of intrinsic fluorescence was observed upon complete chemical induced unfolding. The observed red-shift must be due to tryptophan residues being replaced from the relatively less polar interior of the protein to polar solvent upon unfolding. Chemical denaturation thus completely unfolds the protein into its inactive and linear form unlike thermal or acid denaturation.

The C_m_ value for enzymatic activity was found to be 1.85 and 2.7 M for GdHCl and urea, respectively. The free energy of protein unfolding (ΔG_0_) and rate constant of chemical induced unfolding (K_obs_) was also calculated as given in the methods section using Eq (2) and Eq (3) (Fig 5C and 5D). Half-Chevron plot was constructed from the values of unfolding kinetic rate constants (K_*ui*_) plotted against denaturant concentration, where rate constant of unfolding (K_obs_) equals the value of the y intercept. The C_m_ value for GdHCl denatured mung bean α-amylase was observed to be 1.59 M at pH 5.5, whereas for soybean α-amylase it was found to be around 0.72 M at pH 7.4 [36, 37], indicating that wheat α-amylase is more stable. The transition midpoints by far-UV CD and fluorescence emission are summarized in Table 1 indicating non-spontaneity of the unfolding process.

**Table 1:** Thermodynamic and kinetic parameters for unfolded α-amylase.

Kinetic parameter	GdHCl denaturation		Urea denaturation	
	Far-UVCD	Fluorescence	Far-UVCD	Fluorescence
**ΔG_0_ (kcal/mol)**	0.96	0.923	1.35	1.92
**log K_obs_**	-0.12	-0.6	-0.99	-1.4
**C_m_ (M)**	1.22	1.71	2.0	3.23

## Conclusion

The physico-chemical characteristics of wheat α-amylase have been elucidated to gain insight into the structure-function relationship of the enzyme in view of its stability and conformational transitions toward temperature, pH and chemical denaturants. In the far-UV region, native α-amylase revealed two well-resolved negative peaks at 208 and 222 nm with prominence at 208 nm indicating structural integrity and mixed α-helices and β-sheets (Fig 2A). The low content of secondary structure implied flexible glycine-rich loops in the enzyme reminiscent of mammalian α-amylases and different from insect α-amylases, allowing insect α-amylases as targets of appropriate inhibitors for pest control. This allows an opportunity to create transgenic plants that can express and present specific inhibitors to insect α-amylases during pest attack. The multiple tyrosine and tryptophan side chains in the protein with their characteristic fluorescence properties promises feasible investigation of the local conformational transitions (and thus global three-dimensional structure from combined data) in the enzyme in detail. The denaturant induced investigation of biophysical and catalytic properties of the enzyme revealed that the protein has high stability compared to other similar enzymes.

**Fig 6:**
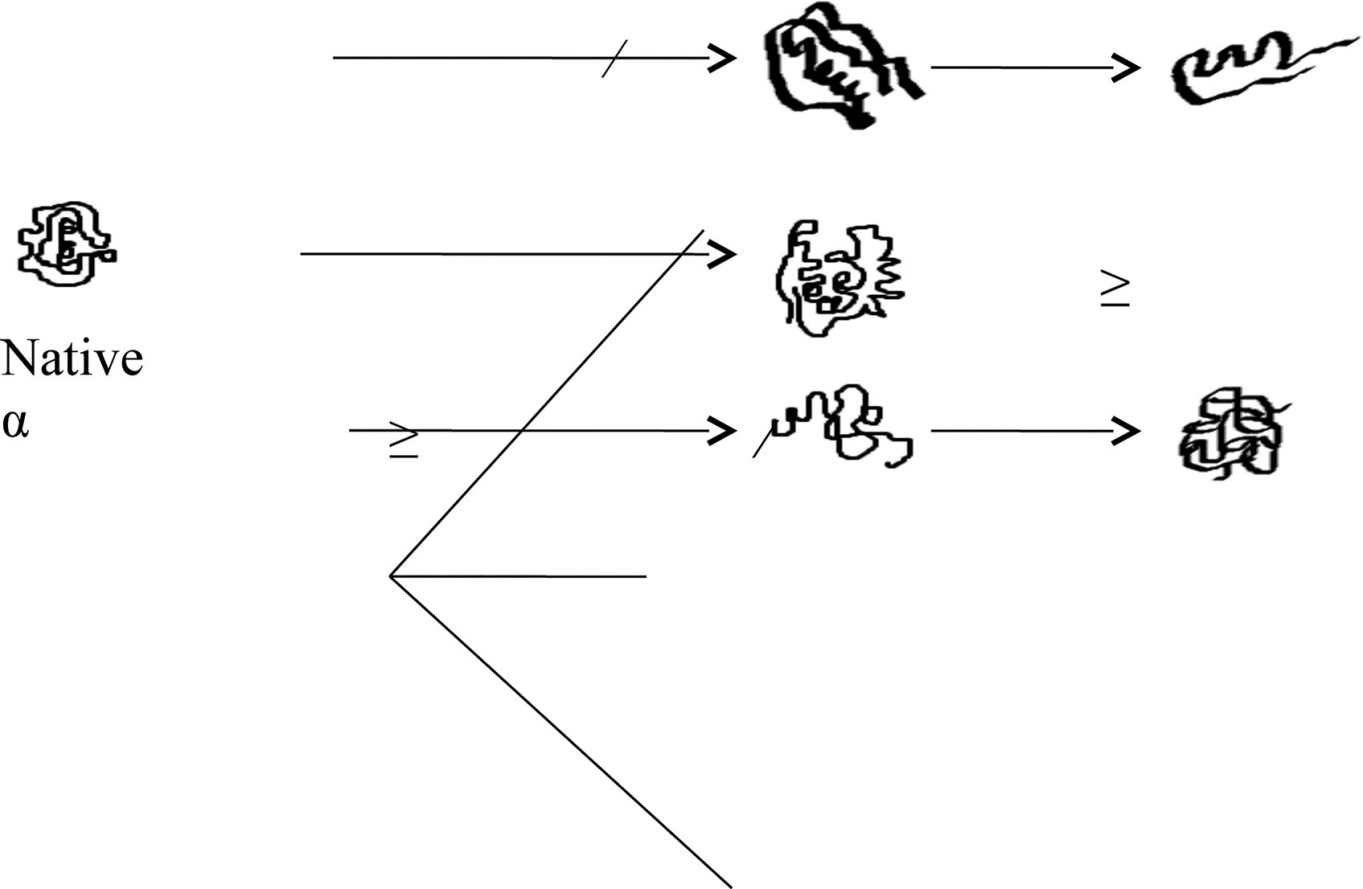
Schematic representation of possible unfolding pathways undertaken by α-amylase during treatment with different denaturants.

α-Amylase demonstrated environment dependent variation in unfolding pathways in accordance to “folding funnel” mechanism [12] of protein folding. The mechanistic scheme of unfolding for α-amylase appears to be a complex process with a number of spectroscopically distinct intermediate states (Fig 6). These conformations were either transient or stable and populated under appropriate equilibrium folding conditions. The high structural integrity of the protein disappeared with melting of secondary structures, activity loss and red-shift of fluorescence emission at 4 M GdHCl or 6 M urea, indicating a rather more heterogeneous scrambled structure. The fluorescence spectrum of the completely unfolded enzyme in 6 M GdHCl remains similar in shape, but the emission maximum shifts from 340 to 355 nm with a 66% decrease in the fluorescence intensity, indicating that more tryptophan residues of α-amylase are exposed to a polar environment.

The acid-induced unfolding transition of α-amylase as monitored by measurement of far-UV CD is cooperative and appears to involve a conversion to somewhat collapsed unfolded state (acid-unfolded state) with some residual secondary structure at pH 2.0. This discrete intermediate (Fig 6) opens up a new dimension for research with the possibility of obtaining new insight in similar classes of enzymes. Chemical induced denaturation leads to complete unfolding of the enzyme through transient intermediates which do not accumulate in measurable yields, but is evident from non-coincidental transition curves. These results were also different from those indicating two-state unfolding mechanism in soybean amylase. Thermal investigations revealed that denaturation was initiated at 75°C, followed by immediate aggregation, which could not be monitored by CD and fluorescence. Thermal denaturation thus indicated the presence of yet another kind of transient intermediate state.

The investigation has also provided multiple evidence of the fact that certain domains of the enzyme, especially its catalytic domain, are more stable than other domains (domain C and B). As such the protein displayed partial loss of secondary structure and local changes in fluorophore conformation even before loss of enzymatic activity. It is possible that loosening of certain domains is required for the enzyme to gain maximum enzymatic activity. This may explain its optimum pH for catalysis to be 5, since at this pH the structural compactness of the enzyme seems to be less than at physiological pH of 7. Detailed knowledge of such structural conformation may allow enhancing the catalytic activity of the enzyme by protein engineering techniques.

This work reiterates that while amino acid sequence are determinants of the final native conformation of a protein, the environment dictates the pathway that a protein would take to fold and attain the three-dimensional conformation required for its physiological role. This probably allows the protein to fold and function in even challenging, environment which may differ from its ambient environment under certain circumstances. This ability might have also helped proteins to survive through the evolutionary selection pressure. In this work, α-amylase diverged from the classical two-state folding mechanism and hence additional study involving the understanding of stability factors may be helpful in further development of enzymes/recombinant protein with improved stability for biotechnological applications.

## Supporting Information

S1 Text Sequence analysis of α-amylase. (DOCX)
