# Correction: Heat, Acid and Chemically Induced Unfolding Pathways, Conformational Stability and Structure-Function Relationship in Wheat α-Amylase

**DOI:** 10.1371/journal.pone.0132764

**Published:** 2015-07-13

**Authors:** Kritika Singh, Manish Shandilya, Suman Kundu, Arvind M. Kayastha

There is an error in Fig 6. Please view the correct Fig 6 here.

**Fig 6:**
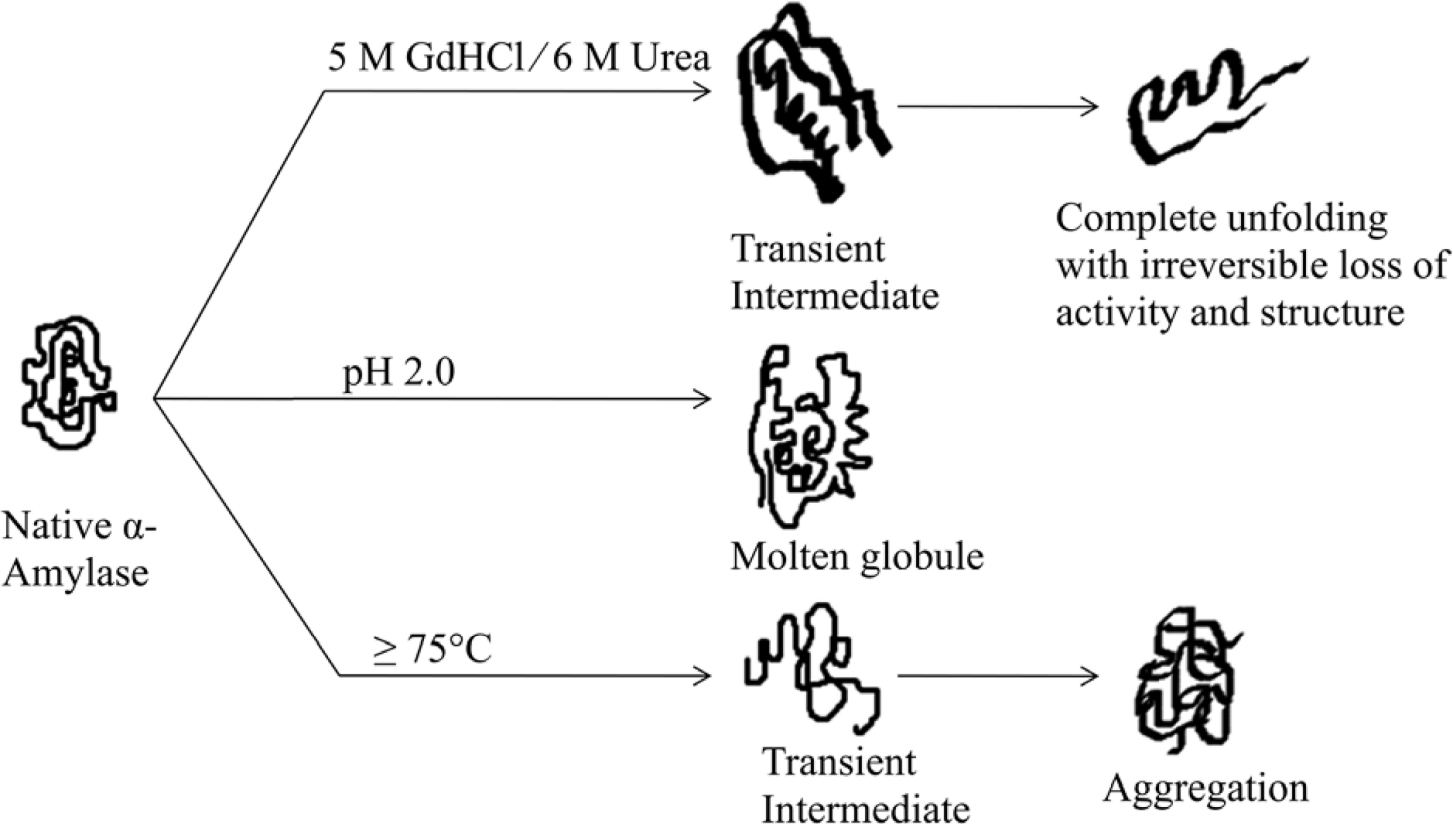
Schematic representation of possible unfolding pathways undertaken by α-amylase during treatment with different denaturants.
