# Supplementary material for: Heat, Acid and Chemically Induced Unfolding Pathways, Conformational Stability and Structure-Function Relationship in Wheat α-Amylase
Source: PLoS One. 2015 Jun 8;10(6):e0129203. doi: 10.1371/journal.pone.0129203 (PMC4460087; doi:10.1371/journal.pone.0129203)
Supplement: S1 Text — (DOCX) [file pone.0129203.s001.docx]

S1

Multiple sequence alignment: A full-length peptide sequence of wheat α-amylase (Accession CAA29252.1) was used for BLAST (Basic Local Alignment Search Tool) using National Centre for Biotechnology Information website (http://blast.ncbi.nlm.nih.gov). A comparison of sequence identity of wheat α-amylase to other known α-amylase (*Hordeum vulgare, Bacilus licheniformis, Aspergillus oryzae*) was performed using ClustalW.

gi|Triticum ---MGKHSATLCGLLVVVLCLASSLAQAQILFQG--FNWESWKT---------------- 39

gi|Hordeum ---MGKN-GNLCCFSLLLLLLAGLASGHQVLFQG--FNWESWKQ---------------- 38

gi|BLA MKQQKRLYARLLTLLFALIFLLPHSAAAAANLNGTLMQYFEWYMPN-------------- 46

gi|A.oryzae -----MMVAWWSLFLYGLQVAAPALAATPADWRSQSIYFLLTDRFARTDGSTTATCNTAD 55

. : : : .. : :

gi|Triticum ---QGGWYKFMQGKVEEIASTGATHVWLPPPSQ--------SVSPEGYLPGQLYNLN--- 85

gi|Hordeum ---SGGWYNMMMGKVDDIAAAGVTHVWLPPPSH--------SVSNEGYMPGRLYDIDA-- 85

gi|BLA ---DGQHWKRLQNDSAYLAEHGITAVWIPPAYKGT------SQADVGYGAYDLYDLGEFH 97

gi|A.oryzae RKYCGGTWQGIIDKLDYIQGMGFTAIWITPVTAQLPQTTAYGDAYHGYWQQDIYSLN--- 112

* :: : .. : * * :*:.* . : ** :*.:.

gi|Triticum ------SKYGSGADLKSLIQAFRGKNISCVADIVINHR-CADKKDGRGVYCIFEG----- 133

gi|Hordeum ------SKYGNAAELKSLIGALHGKGVQAIADIVINHR-CADYKDNRGIYCIFEG----- 133

gi|BLA QKGTVRTKYGTKGELQSAIKSLHSRDINVYGDVVINHKGGADATEDVTAVEVDPADRNRV 157

gi|A.oryzae ------ENYGTADDLKALSSALHERGMYLMVDVVANHMGYDGAGSSVDYSVFKP------ 160

:**. :*:: ::: :.: *:* ** . .. .

gi|Triticum ----------------GTSDNRLDWGPDEICSDDTKYSNGR-----------------GH 160

gi|Hordeum ----------------GTSDGRLDWGPHMICRDDTKYADGT-----------------AN 160

gi|BLA ISGEHLIKAWTHFHFPGRGSTYSDFKWHWYHFDGTDWDESRKLNRIYKFQGKAWDWEVSN 217

gi|A.oryzae ---------------FSSQDYFHPFCLIQNYEDQTQVEDCW------------------- 186

. . : * *. :

gi|Triticum RDTGGGFDAAPDIDHLNPRVQRELSAWLNWLKTDLGFDGWRLDFAKGYSAAMAKIYVDNS 220

gi|Hordeum LDTGADFAAAPDIDHLNDRVQRELKEWLLWLKSDLGFDAWRLDFARGYSPEMAKVYIDGT 220

gi|BLA ENGNYDYLMYADIDYDHPDVAAEIKRWGTWYANELQLDGFRLDAVKHIKFSFLRDWVNHV 277

gi|A.oryzae --LGDNTVSLPDLDTTKDVVKNEWYDWVGSLVSNYSIDGLRIDTVKHVQKDFWPGYNKAA 244

. . .*:* : * * * .: :*. *:* .: . : : .

gi|Triticum -----KPAFVVGELYD---------------RDRQLLANWVRGVGGPATA---FDFPTKG 257

gi|Hordeum -----SPSLAVAEVWDNMATGGDGKPNYDQDAHRQNLVNWVDKVGGAASAGMVFDFTTKG 275

gi|BLA REKTGKEMFTVAEYWQN---------------DLGALENYLNKTNFNHSV---FDVPLHY 319

gi|A.oryzae G------VYCIGEVLDG---------------DPAYTCPYQNVMDGVLNY------PIYY 277

:.* : . : . . .

gi|Triticum VLQEAVQGDLGRMRGSDGKAPGMIGWMPEKTVT--FIDNHDTGSTQRLWP-FPSDKVMQG 314

gi|Hordeum ILNAAVEGELWRLIDPQGKPPDVMGWWPAKAVT--FVDNHDTGSTQPMWP-FPSDKVMQG 332

gi|BLA QFHAASTQGGGYDMRKLLNG-TVVSKHPLKSVT--FVDNHDTQPGQSLESTVQTWFKPLA 376

gi|A.oryzae PLLNAFKSTSGSMDDLYNMINTVKSDCPDSTLLGTFVENHDNPRFASYTN--DIALAKNV 335

: * : . * .:: *::***.

gi|Triticum YAYILTHP-GIPCIFYDHVFDWK--------------------LKQEITALATVRSRNGI 353

gi|Hordeum YAYILTHP-GTPCIFYDHFFNWG--------------------FKDEIAALVAIRKRNGI 371

gi|BLA YAFILTRESGYPQVFYGDMYGTKGDSQRE-----------IPALKHKIEPILKARKQYAY 425

gi|A.oryzae AAFIILND-GIPIIYAGQEQHYAGGNDPANREATWLSGYPTDSELYKLIASANAIRNYAI 394

*:*: . * * :: .. :: . . .

gi|Triticum HPGS------TLDILKAEGDLYVAK-IGGKVITKIG----SRYNIGD-NVIPSGFKIAAK 401

gi|Hordeum TATS------ALEILMHEGDAYVAE-IDGKVVVKIG----TRYDVG--AVIPAGFATSAH 418

gi|BLA GAQHDYFDHHDIVGWTREGDSSVAN-SGLAALITDGPGGAKRMYVGRQNAGETWHDITGN 484

gi|A.oryzae SKDTGFVTYKNWPIYKDDTTIAMRKGTDGSQIVTILSNKGASGDSYTLSLSGAGYTAGQQ 454

: : : . : . : . :

gi|Triticum GNNYC-----VWEKSGL---------------------------- 413

gi|Hordeum GKD------------------------------------------ 421

gi|BLA RSEPV-----VINSEGWGEFHVNGGSVSIYVQR------------ 512

gi|A.oryzae LTEVIGCTTVTVGSDGNVPVPMAGGLPRVLYPTEKLAGSKICSSS 499

.:

Number of aromatic amino acid residues present in α-amylase from following species:

*Hordeum vulagare*

Tryptophan –15

Tyrosine – 15

Phenylalanine –16

*Bacillus licheniformis*

Tryptophan –17

Tyrosine – 31

Phenylalanine –22

*Aspergillus oryzae*

Tryptophan –12

Tyrosine – 35

Phenylalanine –14
